# Near-Infrared Fluorescent Hydroxyapatite Nanoparticles for Targeted Photothermal Cancer Therapy

**DOI:** 10.3390/pharmaceutics15051374

**Published:** 2023-04-29

**Authors:** Gayoung Jo, Yoonbin Park, Min Ho Park, Hoon Hyun

**Affiliations:** 1 Department of Biomedical Sciences, Chonnam National University Medical School, Hwasun 58128, Republic of Korea; 2 BioMedical Sciences Graduate Program (BMSGP), Chonnam National University, Hwasun 58128, Republic of Korea; 3 Department of Surgery, Chonnam National University Medical School and Hwasun Hospital, Hwasun 58128, Republic of Korea

**Keywords:** photothermal therapy, near-infrared fluorescence imaging, hydroxyapatite, P800SO3, targeted nanoparticles

## Abstract

Near-infrared (NIR) fluorophores have attracted great attention due to their excellent optical and photothermal properties. Among them, a bone-targeted NIR fluorophore (named P800SO3) contains two phosphonate groups, which play important roles in binding with hydroxyapatite (HAP) as the main mineral component of bones. In this study, biocompatible and NIR fluorescent HAP nanoparticles functionalized with P800SO3 and polyethylene glycol (PEG) were readily prepared for tumor-targeted imaging and photothermal therapy (PTT). The PEGylated HAP nanoparticle (HAP800-PEG) demonstrated improved tumor targetability with high tumor-to-background ratios (TBR). Moreover, the HAP800-PEG also showed excellent photothermal properties, and the temperature of tumor tissue reached 52.3 °C under NIR laser irradiation, which could completely ablate the tumor tissue without recurrence. Therefore, this new type of HAP nanoparticle has great potential as a biocompatible and effective phototheranostic material, which enables the use of P800SO3 for targeted photothermal cancer treatment.

## 1. Introduction

Recently, photothermal therapy (PTT) has been widely used as one of the most promising approaches for more effective cancer therapy without significant side effects [1,2,3]. Since PTT is a non-chemotherapeutic tumor treatment, it is considered relatively safe compared with the risk of anticancer drugs from the chemotherapy. In order to better apply the PTT strategy, various types of PTT agents have been extensively developed depending on the nanosized materials, including noble metal-, carbon-, and synthetic organic polymer-based nanoparticles [4,5,6,7,8,9]. PTT agents should have strong absorption in the traditional near-infrared (NIR) region (650–950 nm) for efficiently converting light energy into thermal energy to induce hyperthermia, leading to cancer cell death [10,11,12,13,14,15,16]. Although many different types of PTT agents have shown great promise in cancer treatment, the optimization of nanoparticles is still required to overcome the limitations of nanoagent-based PTT, such as low tumor targetability, short tumor retention, poor biocompatibility, potential long-term toxicity, and complicated synthetic processes [17,18,19].

To address biosafety issues, hydroxyapatite (Ca_10_(PO_4_)_6_(OH)_2_, HAP), a hydroxylated calcium phosphate-based biomaterial, has been previously used as a preferable drug carrier because of its good biocompatibility and biodegradability, strong biological activity, and non-mutagenicity [20,21]. HAP nanoparticles can be absorbed into the body and completely excreted from the body after systemic administration, leading to low systemic toxicity [22]. Additionally, HAP nanoparticles have also been found to show certain prohibitory effects on several types of cancer cells including hepatoma, melanoma, breast cancer cells, osteosarcoma cells, gastric cancer cells, and glioma cells, depending on the size of HAP nanoparticles [23,24,25,26]. Typically, the lack of functional groups on the surface of HAP nanoparticles has limited their applicability in vivo. Before being used as a PTT agent, the surface of HAP nanoparticles should be modified to allow for covalent conjugation with photosensitizers [27,28]. Moreover, synthetic amphiphilic polymers (e.g., poly(N-isopropylacrylamide) and poly(L-phenylalanine)) or natural hydrophilic polymers (e.g., chitosan and hyaluronic acid) have been grafted onto the surface of HAP nanoparticles to improve dispersion stability in water [29]. By altering the surface chemical properties, the strong agglomeration tendency of the HAP nanoparticles can also be prevented. To simplify the complex surface modification of the HAP nanoparticles, we proposed a novel strategy that enables a single small NIR fluorophore to bind on the surface of HAP nanoparticles through a facile one-step coating process without any chemical reactions.

P800SO3, a phosphonated heptamethine NIR fluorophore, targets bone tissue without conjugation with bone-targeting ligands such as bisphosphonate drugs [30]. P800SO3 is designed based on the structure-inherent targeting strategy by incorporating phosphonate moieties into the chemical structure of heptamethine cyanine backbone for bone-specific imaging. Since the P800SO3 NIR fluorophore have a strong affinity for HAP, the simultaneous imaging and targeting capabilities of P800SO3-bound HAP (HAP800) could be further applied for effective photothermal cancer treatment. To enhance the tumor accumulation of HAP800, the non-covalent attachment of biocompatible and hydrophilic polyethylene glycol (PEG) on the surface of HAP800 can be considered for prolonging blood circulation and reducing immune clearance [31,32]. Hence, we hypothesized that HAP could assist the tumor-targeted delivery of P800SO3 via the enhanced permeability and retention (EPR) effect, as well as reduced reticuloendothelial system (RES) uptake, owing to noncovalent PEG coating. That is, HAP could help to enhance the tumor accumulation of P800SO3 and facilitate the phototherapeutic performance of P800SO3 during photothermal treatment.

In this present study, a new type of HAP nanoparticles with enhanced tumor accumulation was readily created using HAP800 coated with the PEG (HAP800-PEG). The P800SO3 and PEG were, respectively, utilized for tumor-targeted NIR fluorescence imaging and effective photothermal cancer treatment. In vivo systemic efficacy of HAP800-PEG was conducted using HT-29 tumor-bearing nude mice to evaluate the tumor targetability and PTT effect. The PEGylated HAP800 nanoparticles demonstrated improved tumor uptake with high tumor-to-background ratios (TBR), which could be useful for targeted photothermal cancer treatment.

## 2. Materials and Methods

### 2.1. Preparation of HAP800, HAP800-BSA, and HAP800-PEG

HAP nanopowder (<200 nm particle size), bovine serum albumin (BSA), and PEG (average MW ≈ 20,000 Da) were purchased from Sigma-Aldrich (St. Louis, MO, USA) and used as received. The phosphonated NIR fluorophore (P800SO3) was synthesized as described previously [30]. For HAP800, 25 mg/mL of HAP nanopowder was dispersed in distilled water (DW, 1 mL), and then 100 μM of P800SO3 was added into the solution. The mixture was incubated with continuous vortexing at ambient temperature for 1 h. Subsequently, the mixed solution was centrifuged at 15,800× *g* and ambient temperature for 10 min. The precipitate was washed thoroughly to remove free P800SO3 with repeated centrifuging (at least 3 times). For HAP800-BSA and HAP800-PEG, the obtained HAP800 was added into the BSA or PEG solutions (10 mg/mL), respectively. The mixtures were incubated with continuous vortexing at ambient temperature for 24 h. Finally, the mixed solution was centrifuged at 15,800× *g* and ambient temperature for 10 min. For the purification of HAP800-BSA or HAP800-PEG mixtures, the unbound BSA or PEG residues in each solution were eliminated using a repeated centrifuging and washing method. The dynamic light scattering (DLS; Nano ZS Zetasizer, Malvern Instruments Ltd., Malvern, UK) measurement was carried out to determine the size distribution profile and zeta potential of the commercial HAP nanoparticles.

### 2.2. Optical Property Measurement

Optical measurement was carried out in phosphate-buffered saline (PBS) at pH 7.4. The absorption spectra of P800SO3, HAP, HAP800, and HAP800-PEG were detected by a fiber optic Flame spectrometer covering UV, Visible, and NIR wavelengths (200–1025 nm, Ocean Optics, Dunedin, FL, USA). The purified P800SO3, HAP800, and HAP800-PEG samples dispersed in PBS (pH 7.4) were used to measure fluorescence emission wavelengths using a SPARK^®^ 10M microplate reader (Tecan, Männedorf, Switzerland).

### 2.3. In Vitro Photothermal Conversion Efficiency

HAP800-PEG (100 μM concentration based on P800SO3) dispersed in PBS (100 μL, pH 7.4) was exposed to laser irradiation at 808 nm (1.0 W/cm^2^). The temperature change was monitored using a thermal imager (FLIR Systems, Wilsonville, OR, USA). The heating and cooling were repeated three times to test the photothermal stability of HAP800-PEG. Based on the equations reported previously [33,34,35], the photothermal conversion efficiency (*η*) of HAP800-PEG was calculated as follows:

From an energy balance in a system, we can describe the total energy balance, as follows:

(1) $$\underset{i}{\sum }m_{i}C_{p,i}\frac{dT}{dt}=Q_{\mathrm{HAP}800}+Q_{s}-Q_{loss}$$

The mass is expressed as *m*. The heat capacity of mixture solvent (water) is expressed as *C_p_*. The solution temperature is expressed as *T*.

The photothermal energy input from the HAP800-PEG nanoparticles is expressed as *Q*_HAP800_. The *Q*_HAP800_ is calculated as follows:

*Q*_HAP800_ = *I*(1 − 10^−*A*λ^)*η* (2)

The laser power density is expressed as *I*. The absorbance of HAP800-PEG at 808 nm is expressed as *A*_λ_. The photothermal conversion efficiency generated from the absorbed light energy to heat energy is expressed as *η*. The thermal energy associated with the light absorbance of the solvent is expressed as *Q_s_*.

The heat energy lost to the surroundings is expressed as *Q_loss_* and can be calculated as follows:

*Q_loss_* = *hA*Δ*T* (3)

The heat transfer coefficient is expressed as *h*. The surface area of the container is expressed as *A*. The temperature change is expressed as Δ*T*. The Δ*T* is defined as *T* − *T_surr_* (*T*: solution temperature, *T_surr_*: ambient temperature of the surroundings). After turning the light source off, *hA* can be measured by the rate of temperature decrease. Then, the combination of Equations (3) and (1) produces Equation (4):

(4) $$\underset{i}{\sum }m_{i}C_{p,i}\frac{dT}{dt}=-Q_{loss}=-hA\Delta T$$

After rearrangement and integration, the following expression for *t* is obtained as follows:

(5) $$t=-\frac{\sum_{i}m_{i}C_{p,i}}{hA}\theta$$

*θ* is defined as the ratio of Δ*T* to Δ*T*_max_, as follows:

(6) $$\theta =\frac{\Delta T}{\Delta T_{\max}}$$

According to the cooling curve, *τ_s_* and heat transfer coefficients (*hA*) can be determined as follows:

*t* = − *τ_s_* ln(*θ*) (7)

At the maximum steady-state temperature, the heat input is equal to the heat output, as follows:

*Q*_HAP800_ + *Q_s_* = *Q_loss_* = *hA*Δ*T*_max_ (8)

where Δ*T*_max_ is the temperature change at the maximum steady-state temperature. Therefore, the photothermal conversion efficiency of HAP800-PEG can be calculated as follows:

(9) $$\eta =\frac{hA\Delta T_{\max}-Q_{s}}{I\left(1-10^{-A\lambda }\right)}$$

### 2.4. HT-29 Xenograft Mouse Model

The in vivo study using mice was carried out according to the animal protocol approved by Chonnam National University Animal Research Committee (CNU IACUC-H-2020-19). We purchased male athymic nude mice (6 weeks old and ≈25 g) from OrientBio (Gwangju, Republic of Korea) to prepare human tumor xenograft models. The human colorectal adenocarcinoma cell line (HT-29) was selected to prepare the xenograft mouse model and purchased from the American Type Culture Collection (ATCC, Manassas, VA, USA). We purchased Roswell Park Memorial Institute (RPMI) 1640 medium (Gibco BRL, Paisley, UK) for maintaining the HT-29 cancer cell line. Antibiotic/antimycotic solution (Welgene, Daegu, Republic of Korea) and 10% fetal bovine serum (FBS, Gibco BRL) were added as supplement to the RPMI medium before starting cultures. The HT-29 cancer cells were incubated in a humidified 5% CO_2_ atmosphere at 37 °C. A 100 μL (1 × 10^6^ cells per mouse) volume of cell suspension in PBS (pH 7.4) was subcutaneously inoculated into the mouse higher right flank. Finally, each fluorescent sample was intravenously administered to mice bearing subcutaneous tumors with an average diameter of 1 cm in size at 8–10 days post-inoculation.

### 2.5. In Vivo Biodistribution and Tumor Imaging

The mice in each treatment group were anesthetized at 4 h post-injection. Subsequently, the tumors and organs (heart, lungs, liver, pancreas, spleen, kidneys, duodenum, and intestine) were excised for NIR fluorescence imaging to confirm the time-dependent biodistribution and clearance of each sample. The FOBI imaging system (CellGenTek, Deajeon, Republic of Korea) was utilized for in vivo NIR fluorescence imaging in real-time. The open-source ImageJ software (National Institutes of Health, Bethesda, MD, USA) was used to quantify the fluorescence intensities on the tumor area and resected organs.

### 2.6. In Vivo Photothermal Therapeutic Efficacy

HAP800, HAP800-PEG, or PBS (100 μL) were administered into the lateral tail veins of the HT-29 tumor-bearing mice. Subsequently, the tumor-bearing mice were anesthetized after 24 h of injection. The 808 nm laser with 1.0 W/cm^2^ power density was irradiated on the tumors. The thermal imager (FLIR Systems) was used to monitor the temperature changes at the tumor area in real time. At 24 h post-irradiation, the mice in each treatment group were anesthetized for collecting tumors to observe the histological changes after the hematoxylin and eosin (H&E) staining process. The tumor growth and body weight of mice in each treatment group were observed for 9 days to evaluate the photothermal therapeutic efficacy and systemic toxicity, respectively. The formula longest diameter × (shortest diameter)^2^ × 0.5 was used to determine the tumor volume.

### 2.7. Histological Analysis

The Nikon Eclipse Ti-U inverted microscope system (Nikon, Seoul, Republic of Korea) was utilized for assessment of histopathological changes in each treatment group, and 4% paraformaldehyde solution was used to fix the resected tumors. The fixed tumor tissues were stored in a deep freezer. Subsequently, the frozen tumor tissues were processed for cryosectioning (e.g., 5–10 μm thickness) and staining with H&E.

## 3. Results and Discussion

### 3.1. Preparation of NIR Fluorescent HAP Nanoparticles

**Figure 1:**
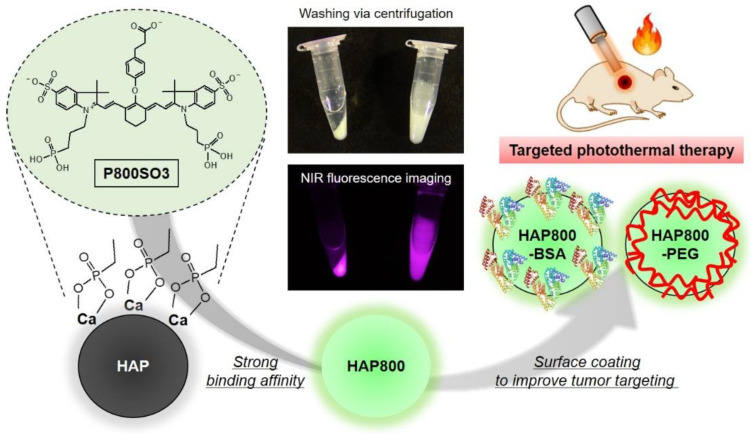
Preparation scheme of NIR fluorescent HAP nanoparticles for targeted NIR fluorescence imaging and PTT.

Previously, most HAP-based nanoparticles were prepared using covalent chemical conjugation with photosensitizer after surface modification of the HAP nanoparticles [27,28]. However, there are several problems during the fabrication process, such as low conjugation efficiency caused by low yield of surface functionalization, biosafety concerns caused by the use of toxic chemicals during the surface modification process, and so on. To address the issues, herein, the bone-tissue-specific NIR fluorophore P800SO3 is especially suitable for preparing NIR fluorescent HAP nanoparticles without additional chemical modification of the HAP surface. As shown in Figure 1, the P800SO3 NIR fluorophore with two phosphonate and sulfonate groups was used as a photosensitizer as well as an imaging agent to form the NIR fluorescent HAP nanoparticles. Although the synergistic effect of sulfonates and phosphonates in the P800SO3 structure for HAP binding is not fully understood, one possible explanation is that the phosphonate groups bind with calcium ions in the surface groups of HAP by acting as a bidentate ligand for calcium binding. Moreover, the NIR fluorescent HAP nanoparticles can be coated with the biocompatible BSA or PEG to improve their water dispersity and stability for prolonged blood circulation, thereby resulting in enhanced tumor accumulation. For the purpose of this study, the P800SO3-bound HAP nanoparticles can serve as a multifunctional phototherapeutic agent for targeted photothermal cancer therapy.

### 3.2. Optical and Size Characterization of HAP Nanoparticles

**Figure 2:**
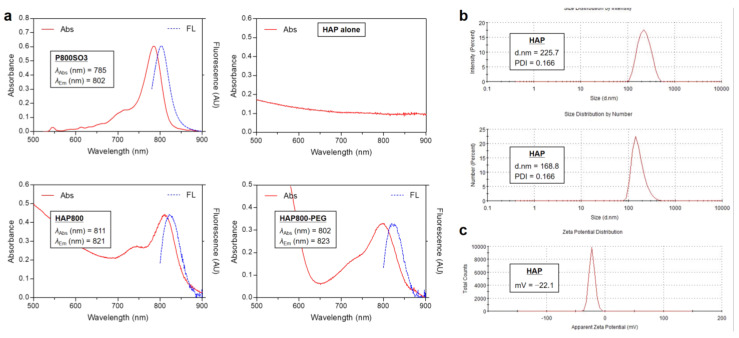
(**a**) Absorption and fluorescence emission spectra of P800SO3, HAP, HAP800, and HAP800-PEG measured in PBS, with a pH of 7.4. (**b**) Size distributions and (**c**) zeta potential of HAP nanoparticles measured in DW.

The NIR absorption and fluorescence emission spectra of P800SO3, HAP, HAP800, and HAP800-PEG are shown in Figure 2a. Similar to the previous report, the P800SO3 alone exhibited an absorption peak at 785 nm and fluorescence emission maximum at 802 nm in PBS, with a pH of 7.4. Interestingly, the HAP800 and HAP800-PEG displayed red-shifted absorption peaks at 811 and 802 nm and fluorescence emission maximum at 821 and 823 nm, compared with that of P800SO3 alone, indicating that HAP800 was successfully prepared by simple mixing of P800SO3 and HAP nanoparticles. NIR fluorescence imaging was also used to reconfirm whether P800SO3 was bound to HAP nanoparticles, as shown in Figure 1. The excitation wavelength of the P800SO3 and HAP800 solutions to obtain the fluorescence emission spectra was 750 nm. The absorption peak of HAP800 was different from that of P800SO3 alone, and the obvious peak shift could be observed. There are two commonly used NIR lasers for PTT, which are 660 and 808 nm wavelengths, because of their deep tissue penetration. Since the absorption peaks of P800SO3 and HAP800-PEG appeared at 785 and 802 nm, respectively, the optical property of HAP800-PEG is more suitable for PTT applications after combining with the 808 nm NIR laser irradiation system.

For DLS analysis, the size distribution of the commercially available HAP nanoparticles was determined using 1 mg/mL of HAP nanopowder in DW. Based on the guideline of DLS measurement provided by the instrument company, it is difficult to measure the colored or fluorescing samples because laser light cannot be absorbed in colored or fluorescent solutions. Hence, the DLS measurement of HAP nanoparticles was performed using the HAP alone before binding with the P800SO3 NIR fluorophore. The sizes of HAP were determined as 225.7 nm (intensity-based analysis) and 168.8 nm (number-based analysis), respectively (Figure 2b). This result confirms that the average size of HAP nanoparticles is consistent with the given information (<200 nm particle size) of the commercial HAP nanopowder. Additionally, the surface zeta potential of HAP was found to be −22.1 mV (Figure 2c). Typically, it is known that the zeta potential of bone-derived HAP showed a negative value, which promotes attachment and proliferation of bone cells [36].

### 3.3. Time-Dependent In Vivo Tumor Imaging and Biodistribution

**Figure 3:**
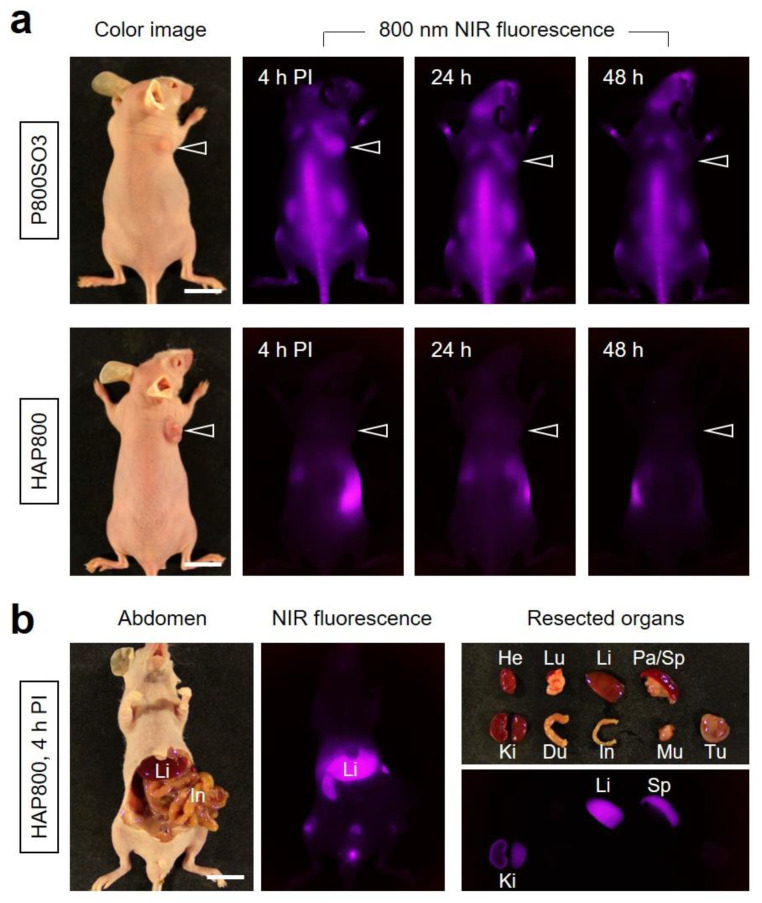
In vivo HT-29 tumor targeting efficiency and biodistribution of P800SO3 and HAP800. (**a**) NIR fluorescence imaging 48 h after injection of P800SO3 and HAP800, respectively. The arrowheads indicate the tumor area. Scale bars = 1 cm. (**b**) Resected organs and tumor tissue were imaged at 4 h post-injection of HAP800. Abbreviations: Du, duodenum; He, heart; In, intestines; Ki, kidneys; Li, liver; Lu, lungs; Mu, muscle; Pa, pancreas; Sp, spleen; Tu, tumor; PI, post-injection. The representative images were selected from each treatment group.

The in vivo NIR fluorescence imaging was performed to investigate the tumor accumulation of HAP800 using the HT-29 xenograft tumor model. The P800SO3 alone and HAP800 solutions dispersed in PBS (100 μM concentration of P800SO3) were intravenously administered to the tumor-bearing mice (Figure 3a). Subsequently, the tumor mice were continually monitored at different time points for 48 h to determine the optimal accumulation time of the tumor site. Similar to the previous report, the P800SO3 alone showed high uptake in bone tissue until 48 h, while the fluorescence intensity of tumor gradually decreased within 24 h post-injection of P800SO3. Although the P800SO3 was accumulated in tumor tissue at 4 h post-injection with similar fluorescence intensity of bone tissue, the shoulder bones close to the tumor could be affected by NIR laser irradiation during photothermal cancer treatment. It is important to consider the prevention of normal tissue damage for safe and accurate PTT application.

Unexpectedly, the tumor treated with HAP800 revealed no significant accumulation within 48 h after injection, and the fluorescence signals of HAP800 were mainly detected in the gastrointestinal tract during 48 h post-injection. According to the fluorescence intensities of resected organs and tumors, the HAP800 was highly accumulated in the liver and mostly eliminated by the intestinal route as a result of hepatobiliary excretion in the body (Figure 3b). This result suggests that the short blood circulation and high RES uptake of HAP800 may affect the poor tumor accumulation. Thus, the surface modification of HAP800 can be considered for prolonging blood circulation and reducing immune clearance to enhance the tumor accumulation of HAP800.

**Figure 4:**
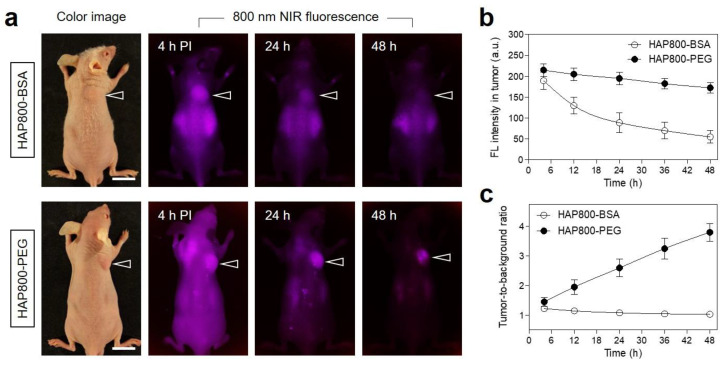
In vivo HT-29 tumor targeting efficiency and biodistribution of HAP800-BSA and HAP800-PEG. (**a**) NIR fluorescence imaging 48 h after injection of HAP800-BSA and HAP800-PEG. The arrowheads indicate the tumor area. Scale bars = 1 cm. (**b**) Time-dependent fluorescence intensities at the tumor sites targeted by HAP800-BSA and HAP800-PEG. (**c**) Tumor-to-background ratio (TBR) of HAP800-BSA and HAP800-PEG were observed for 48 h post-injection. TBR was considered between the fluorescence signals of the tumor and the neighboring area. The representative images were selected from each treatment group.

To improve the tumor targetability of nanoparticles, BSA and PEG are typically used for the surface modification of nanoparticles, in terms of their biocompatibility and tumor targetability. On the basis of the high biocompatibility and long blood circulation of BSA and PEG, HAP800 was, respectively coated with the BSA or PEG to prepare HAP800-BSA and HAP800-PEG nanoparticles for tumor-targeted imaging and PTT application. As shown in Figure 4a, the HAP800-BSA and HAP800-PEG nanoparticles were further investigated using HT-29 tumor-bearing mice to confirm the tumor-specific accumulation. HAP800-BSA and HAP800-PEG were intravenously administered to tumor-bearing mice and the mice were imaged for 48 h post-injection under the NIR fluorescence imaging system. The mice injected with HAP800-BSA showed the high fluorescence in the tumor area until 4 h post-injection. However, the tumor fluorescence gradually decreased after 4 h of injection and mostly disappeared at 48 h post-injection (Figure 4b). Similar to the result of HAP800-BSA, the high fluorescence signal at the tumor site treated with HAP800-PEG was also detected with high background uptake at 4 h after administration. Interestingly, the high fluorescence signal of HAP800-PEG in the tumor was maintained until 48 h of injection. Although the BSA coating on HAP800-BSA played an important role in tumor accumulation through the receptor-mediated endocytosis of albumin, the rate of BSA dissociation from the HAP800-BSA is faster than that of HAP800-PEG. Moreover, HAP800-PEG showed better tumor-targeted imaging with reduced background fluorescence compared to that of HAP800-BSA. After considering the fluorescence intensity and TBR, the PTT performance using HAP800-PEG can be conducted at 24 h post-injection to avoid the unnecessary injury of neighboring normal tissue (Figure 4c). This suggests that the PEGylation of the HAP800 contributed to the enhanced tumor accumulation with reduced background uptake.

### 3.4. In Vitro and In Vivo Photothermal Effects

**Figure 5:**
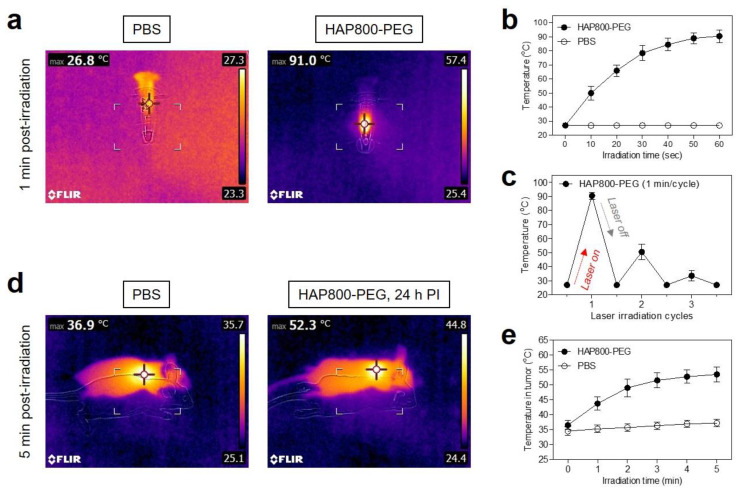
(**a**) In vitro thermal images of the HAP800-PEG and PBS solutions irradiated with an 808 nm laser with 1.0 W/cm^2^ power density for 1 min. The infrared thermal imager was used to monitor the maximum temperature in real time. (**b**) Temperature changes in the HAP800-PEG and PBS solutions were observed for 60 s of laser irradiation. (**c**) Photobleaching test of the HAP800-PEG solution (100 μM) during 3 cycles of laser irradiation. (**d**) In vivo thermal images at the tumor area 5 min post-irradiation of the 808 nm laser with 1.0 W/cm^2^ power density after 24 h of the injections of the HAP800-PEG and PBS solutions. (**e**) Temperature changes of tumors treated with the HAP800-PEG and PBS for 5 min of laser irradiation with 1.0 W/cm^2^ power density. The representative images were selected from each treatment group.

Since HAP800-PEG had a maximum absorption at 802 nm in PBS, an 808 nm wavelength laser was used optimally to perform photothermal conversion of the HAP800-PEG nanoparticles for further in vivo studies. To evaluate the photothermal conversion efficiency of HAP800-PEG, we observed temperature changes in solutions of HAP800-PEG (100 μM concentration of P800SO3) and PBS alone during 1 min irradiation from the 808 nm NIR laser (1.0 W/cm^2^). The temperature change was continuously recorded using a thermal imager in real time (Figure 5a). As shown in Figure 5b, the temperature of the HAP800-PEG solution rapidly increased up to 91 °C under 808 nm laser irradiation for 1 min, while the PBS solution showed no change in temperature under the same irradiation condition. The temperature of the HAP800-PEG solution reached about 78 °C within the first 30 s of irradiation, and then the temperature was maintained up to 90 °C for the next 30 s of irradiation. According to the previous method [35], the photothermal conversion efficiency (*η*) of HAP800-PEG was calculated as 34.7%. The *η* value of HAP800-PEG is similar to that of the polymethine cyanine fluorophores reported previously [33,34]. This result demonstrates that HAP800-PEG can be employed as a promising PTT agent for effective photothermal cancer therapy. Moreover, the photothermal stability of HAP800-PEG was examined for three cycles of laser irradiation using the 100 μM of HAP800-PEG solution (Figure 5c). As expected, the temperature of the HAP800-PEG solutions diminished rapidly in the second cycle. In the third cycle, the temperature of the HAP800-PEG solutions mostly returned to the ambient temperature after the continuous laser irradiation. This result revealed that the polymethine cyanine backbone of P800SO3 can be damaged by the concentrated NIR light, which is consistent with previous reports [33,34]. After confirming the optimal accumulation time of the tumor site, we performed the in vivo PTT treatment at 24 h post-injection of HAP800-PEG. A total of 100 μM of HAP800-PEG or PBS alone was intravenously administered into the HT-29 tumor-bearing mice. At 24 h post-injection, the mice were exposed to the 808 nm NIR laser with 1.0 W/cm^2^ power density for 5 min (Figure 5d). The power density of the 808 nm laser (1.0 W/cm^2^) was previously optimized to avoid unnecessary injury in normal tissue due to the laser power alone [37]. The tumor temperature of the PBS group showed no significant change after 5 min of exposure to the 808 nm NIR laser, whereas that of the tumor in the HAP800-PEG group revealed a high temperature change (52.3 °C) under the same irradiation condition. Most importantly, the temperature of the tumor treated with HAP800-PEG increased by approximately 50 °C after 2 min of laser irradiation, and then the tumor temperature was maintained up to 53 °C for the next 3 min of laser irradiation, which is sufficient temperature to induce tumor necrosis (Figure 5e). Thus, these results suggest that the tumor-targeted HAP800-PEG can be a promising PTT agent for in vivo photothermal tumor ablation.

### 3.5. In Vivo Photothermal Therapeutic Efficacy

**Figure 6:**
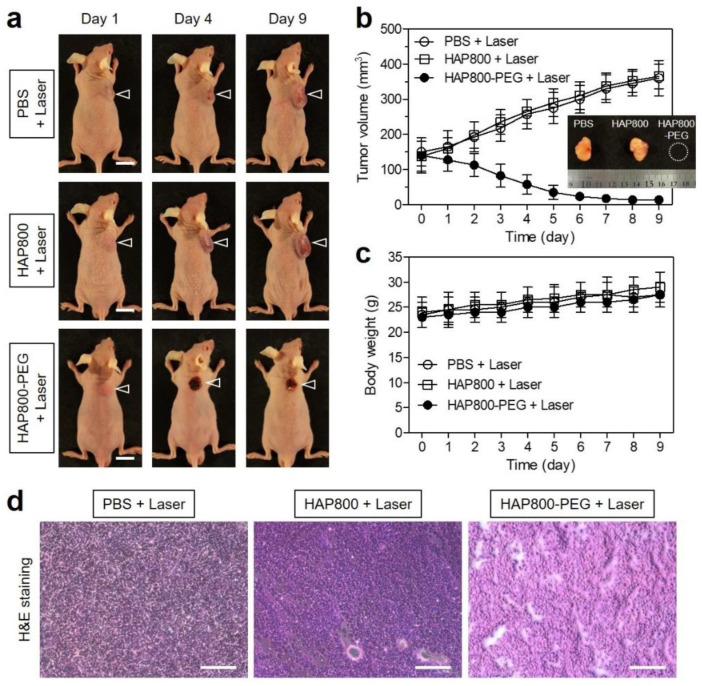
(**a**) Photos of HT-29 tumor-bearing mice observed for 9 days in each treatment group. The tumors were irradiated with an 808 nm laser with 1.0 W/cm^2^ power density for 5 min after 24 h of injections of PBS, HAP800, or HAP800-PEG. The arrowheads indicate the tumor area. Scale bars = 1 cm. (**b**) Tumor growth rates (inset: the gross photo of tumors harvested from each treatment group at day 9) and (**c**) body weights of each treatment group were observed during the course of different treatments. (**d**) Histological observation of tumor slides after staining with H&E in each treatment group. Scale bars = 100 μm. The representative images were selected from each treatment group.

As shown in Figure 6a, we observed the volume changes in tumors in the different groups of mice during the course of treatment to evaluate the phototherapeutic efficacy. As expected, the tumors of the HAP800 and laser-treated group showed no phototherapeutic effect for 9 days, which is similar to that of the PBS and laser-treated group. Importantly, the tumors in the HAP800-PEG and laser-treated group revealed a significant phototherapeutic effect with complete tumor ablation and no recurrence during the period of treatment after NIR laser irradiation (Figure 6b). Hence, this result demonstrated that the enhanced tumor accumulation and lower background uptake of HAP800-PEG are highly important for safe and effective PTT because there was no normal tissue injury from the laser irradiation alone. During the treatment period, the mice in each treatment group showed no significant change in body weight. The mice in all treatment groups exhibited a slow increase in body weight, which indicates the good in vivo biocompatibility of HAP800 and HAP800-PEG nanoparticles (Figure 6c). Finally, the remarkable antitumor capability of HAP800-PEG was reconfirmed by H&E staining (Figure 6d). The tumor tissues were collected from each group after 24 h of different treatments and used for histological observation. As expected, the tumor tissues obtained from the PBS and HAP800 treatment groups exhibited no tumor cell damage. Compared with the control groups, the treatment of HAP800-PEG and laser irradiation caused significant tumor cell damage, accompanied by shrinkage necrosis, which is consistent with the result of the in vivo antitumor effect. Those results suggest that the HAP800-PEG nanoparticles can be successfully used as a biocompatible PTT agent for safe and effective cancer treatment.

## 4. Conclusions

In summary, a new type of HAP nanoparticles was readily prepared and utilized for tumor-targeted imaging and accurate photothermal treatment. Herein, the two important components of HAP nanoparticles were employed simultaneously to enhance the tumor-specific accumulation with high TBR values: P800SO3 for playing the leading role in the photothermal effect as well as NIR fluorescence imaging, and PEG for improving the tumor uptake and retention without nonspecific background uptake. Moreover, under NIR laser irradiation, HAP800-PEG could not only show good photothermal conversion efficiency but also completely ablate the tumor tissue without recurrence and side effects in HT-29 tumor-bearing mice. Therefore, the biocompatible HAP nanoparticles developed in this study have the great potential to be a safe and effective phototherapeutic nanoagent for future clinical applications.

## Data Availability

Not applicable.
